# Incidence of and improvement in inappropriate parental behaviors of mothers with young children: a retrospective cohort study conducted in collaboration with a local government

**DOI:** 10.1186/s13690-021-00558-8

**Published:** 2021-03-17

**Authors:** Takehiro Arai, Aya Goto, Mitsuko Komatsu, Seiji Yasumura

**Affiliations:** 1 Faculty of Education, Art and Science, Yamagata University, 1-4-12 Kojirakawa, Yamagata City, Yamagata 990-8560 Japan; 2 Department of Public Health, School of Medicine, Fukushima Medical University, 1 Hikarigaoka, Fukushima City, Fukushima 960-1295 Japan; 3 Center for Integrated Science and Humanities, Fukushima Medical University, 1 Hikarigaoka, Fukushima City, Fukushima 960-1295 Japan; 4 Fukushima City Health and Welfare Center, 10-1 Moriaicho, Fukushima City, Fukushima 960-8002 Japan

**Keywords:** Cohort studies, Parenting, Financial support, Child abuse, Japan

## Abstract

**Background:**

Inappropriate parental behaviors of mothers toward young children require further study; few epidemiological studies have utilized longitudinal analysis of region-based cohorts. This study examined the frequency of incidence of and improvements in inappropriate parental behaviors of mothers with young children and related factors.

**Methods:**

Among the mothers who underwent a checkup in Fukushima City in 2017, 586 mothers with data from 4-, 18-, and 42-month-old checkups were included in analysis. In this retrospective cohort study, an anonymous database was created by transcribing and matching health checkup records with questionnaires stored at the city health center. Data were analyzed using chi-square tests and logistic regression analysis, using the SPSS Ver.20.0.

**Results:**

In 28.5% of mothers, inappropriate parental behaviors were not reported in the 18-month-old data but were reported in the 42-month-old data. In 3.8%, inappropriate parental behaviors were reported in the 18-month-old data but were not reported in the 42-month-old data. The most common inappropriate parental behavior reported was “yelling at the child using emotional words” (18-month-old data, 16.2%; 42-month-old data, 39.5%). Mothers with financial difficulties were 2.19 times (95%CI: 1.13–4.26) more likely to begin inappropriate parental behaviors between 18 and 42 months. Improvements in parental behaviors were significantly higher in mothers under 30 years old (*p* = 0.03).

**Conclusions:**

It is necessary to identify mothers with financial difficulties early and to examine how to provide childcare and financial support from a local government at the time of child health checkup.

## Background

Child abuse is a globally recognized serious health issue among children as it not only directly threatens their health and safety but also results in lifelong impediments, such as growth and developmental issues and socioeconomic disparities [1–4]. Corporal punishment and abusive language can seriously affect a child’s brain development [5, 6]. Along with negative biological consequences, maltreated children may experience highly undesirable effects including mental health problems and increase in antisocial behavior as well as aggression [1–4]. Moreover, it has been reported that child abuse has negative effects on various milestones in life, such as attending school [7], which can cause the cycle of poverty to be repeated to the next generation [8].

For this reason, various countries have reported their abuse prevention methods [9–11]. In Western countries, the effects of interventions for preventing or reducing have been examined. Many reports have concluded that preventative interventions focused on increasing the confidence of both parents are highly effective, as well as cognitive behavioral therapy and home visit interventions that improve parenting skills or the parents’ life skills, resolve the parents’ mental health problems, or provide emotional and social support for mothers [12–15]. Similarly in East Asia and Pacific regions, reports on the rates of occurrence of child abuse have highlighted the need for preventative measures that consider the circumstances of each country and region [16]. It has been suggested that a comprehensive study of adverse experiences in childhood is needed to come up with policies for this issue [17, 18].

Child abuse is also a serious child health problem in Japan and has long-term social consequences [19]. The number of child abuse consultations handled by the Child Guidance Center in FY2017 was 135,152, which was 3.3 times greater than the previous 10 years, and this number is increasing each year [20]. The “Healthy Parents and Children 21,” a national campaign launched in 2001 for improving maternal and child health, has prioritized the prevention of child abuse beginning in pregnancy and encouraged the country to promote the parenting of children without corporal punishment.

In light of the trend of tolerance for corporal punishment toward children in Japan [21] inappropriate parental behaviors that lead to abuse are reported by a proportion of parents who hit the child, leave the child alone, or ignore the crying child [22]. In addition, it has been reported that the proportion of parents who shake their children or cover their mouths is similar to that in Western countries [23]. Compared to affluent parents, parents with poor social capital have a higher tendency to hit their child with objects or their hands, cover their child’s mouth, or shake their child violently [24]. Young mothers who undergo an unintended pregnancy are at increased risk for perpetrating abuse, especially for covering their child’s mouth [25]. Furthermore, maternal isolation while parenting, postpartum depression after childbirth, and the inability to form attachments to the child can lead to inappropriate parental behavior [26–28]. In Japan, the revised Child Abuse Prevention Law and Child Welfare Law prohibiting domestic corporal punishment will be enforced beginning in April 2020.

To date, most research has utilized cross-sectional study designs, with few longitudinal studies in region-based cohorts [29]. Therefore, in this study performed in collaboration with the government of Fukushima City, longitudinal data obtained from infant health checkups were analyzed. We discuss in detail the incidence of, frequency of improvements in, and factors associated with inappropriate parental behaviors of mothers with infants. In addition, we examine how these findings can be applied to support parenting and childcare.

## Methods

Herein we present a retrospective cohort study. Among 707 children who underwent a health checkup at 42 months of age in Fukushima City from May to September 2017, 586 mothers had all of the required data from 4-, 18-, and 42-month-old checkups and were selected as subjects for analysis. The data used for the analysis were transcribed from the files for these health checkups conducted by Fukushima City, which recorded the health indices of parents and children, as well as parenting behaviors extracted from the Healthy Parents and Children 21 questionnaire. Since these two datasets were stored at the city health center as separate files, they were collated into an anonymous database. The baseline of the retrospective cohort study was the 4-month-old health checkup. The attributes of the participants were ascertained from the questionnaire at the 4-month-old health checkup. The outcome index of inappropriate maternal parenting behaviors was based on the responses to the Healthy Parents and Children 21 items at the time of the 18- and 42-month-old health checkups.

### Analysis items included in the database

For the 4-month-old health checkups, the parents’ ages, employment status, family composition, health status, history of mental illness of the mother, and feelings during pregnancy were transcribed from the health checkup files. Sex, birth order, gestational age at delivery, single or multiple birth, and health status were transcribed from the health checkup files as the attributes of the child. Problems with interpersonal relationships or finances and accessibility to help with childcare or advice about childcare were transcribed as well.

For the 18- and 42-month-old health checkups, the following parenting items were transcribed from the health checkup files and the Healthy Parents and Children 21 questionnaires items and were collated: childcare during the daytime, need for follow-up after the checkup, parental behaviors, parental smoking, whether they want to continue raising their child in the region, whether the father participates in childcare, and the depressive tendencies of the mother.

### Variable definitions

1. The question regarding the mothers’ feelings in relation to the pregnancy was phrased as follows: “How did you feel when you found out about this pregnancy? Please circle one. 1. I was happy 2. I was surprised and happy 3. I was surprised and confused 4. I had difficult feelings 5. I didn’t really think about it.” The responses were classified into “Response 1″ and “Other.”
2. The question regarding the mothers’ physical health was phrased as follows: “How is your physical condition? Please circle all that apply. 1. Good 2. I get tired easily 3. I can’t sleep 4. Not good 5. I have no appetite.” The responses were classified into “Response 1″ and “Other.”
3. The question regarding the mothers’ feelings was phrased as follows: “How are you feeling emotionally? Please circle one. 1. Good 2. Nothing in particular 3. Not good.” The responses were classified into “Response 1″ and “Other.”
4. Inappropriate maternal parental behaviors were identified from the responses to the Healthy Parents and Children 21 questionnaire. The question was, “Did you experience any of the following during your months at home? Please circle all that apply. 1. Enforced too much discipline. 2. Hit my child when I was emotional. 3. Went out while leaving the child alone at home. 4. Did not provide a meal to the child for a long time. 5. Yelled at the child using emotional words. 6. None of the above.” If any of the responses numbered 1 to 5 were chosen, this was deemed as inappropriate parental behavior following the Healthy Parents and Children 21 guidelines.
5. The question regarding perceptions of the local area was phrased as follows: “Do you want to continue raising children in this area in the future? Please circle one. 1. Yes 2. Probably 3. Probably not 4. No.” The responses were classified into “Response 1” and “Other.” Moreover, the question about fathers participating in childcare was phrased as follows: “Does the father of your child take part in childcare? Please circle one. 1. He often does 2. He sometimes does 3. He hardly does 4. I’m not sure.” The responses were classified into “Response 1” and “Other.”
6. The support that the mothers received from those around them was determined based on their responses to two questions with yes/no answer options: “Are there any institutions or people that you can seek advice from when you need consultation?” and “When you are facing a problem, are there any institutions or people whom you can seek support from? (spouse, parent or sibling, friend, neighbor, governmental or non-governmental services, other)” [30].
7. Regarding family issues, in response to the question “Do you have any problems at this moment regarding your family?” it was determined that there were interpersonal problems if the mother responded “yes” to at least one of the following: “differences in parenting approach, difficulty in gaining support for childcare, a lack of conversation, having difficulties with relatives.” Similarly, it was determined that there were financial problems if the subject responded “yes” to at least one of the following: “unstable income, differences in views on finances, loss of employment, job change, gambling, unplanned loan” [31].

### Statistical analysis

The proportion of cases with inappropriate parental behaviors at the time of the 18- and 42-month-old checkups was evaluated, as well as the proportions of changes in inappropriate parental behaviors between the two time points. Regarding the factors related to inappropriate parental behaviors at each timepoint, univariate analysis was conducted followed by multivariate analysis of significant factors (*p* < 0.05). The univariate analysis was followed by multivariate analysis to control the confounding factors associated with inappropriate parental behaviors at the 18-month checkup. Multivariate analysis was performed by entering the factors for which a significant association was found in the univariate analysis, namely, order of birth, feelings in relation to the pregnancy, mother’s physical health, mother’s feelings, interpersonal problems, and financial problems. Most of information or potential confounding factors were extracted from the 4-month-old checkups as indicated in Tables 4 and 5. Furthermore, in the group with new inappropriate parental behaviors that emerged between the 18- and 42-month-old checkups, the risk ratio was calculated, and multivariate analysis was conducted using factors with a statistical significance of *p* < 0.1 by univariate analysis. The covariates entered into the multivariate analysis were financial problems, mother’s feelings, and the number of people to seek cooperation from. In addition, in the group wherein inappropriate parental behaviors improved between the 18- and 42-month-old checkups, the related factors were examined. For univariate analysis, a chi-squared test and Fisher’s exact test were used. For multivariate analysis, binomial logistic analysis was used. Statistical significance was set at *p* < 0.05 for this study. For all statistical analyses, SPSS Ver.20.0 was utilized.

## Results

Among 707 attendants of the 42-month-old checkups during the studied period, we analyzed data of 586 attendants with all of the required data from 4-, 18-, and 42-month-old checkups. Table 1 shows the participants’ characteristics. The median age of mothers was 31.0 years, and 54.1% responded that they were working. The median age of fathers was 33.0 years, and 99.7% responded that they were working. As for child sex, 52.9% of children were boys and 47.1% were girls. Among them, 4.4% were born pre-term and 7.5% underweight.

**Table 1:** Characteristics of participants

	N (%)^a^ or *median (min, max)*
	*N* = 586
Characteristics of the mothers	
Age	*31.0 (19, 53)*
Employment	
Not employed	260 (45.9)
Employed	306 (54.1)
Characteristics of the fathers	
Age	*33.0 (18, 55)*
Employment	
Not employed	2 (0.3)
Employed	573 (99.7)
Characteristics of the children	
Sex	
Male	310 (52.9)
Female	276 (47.1)
Order of birth	
First child	276 (47.1)
Second child or later	310 (52.9)
Gestational age at birth	
Less than 37 weeks	26 (4.4)
37 weeks or more	560 (95.6)
Birth weight	
Less than 2500 g	44 (7.5)
2500 g or more	542 (92.5)

For some items, the total does not add up to the number in the top row due to missing data

^a^The proportion with the parameter as the number of valid responses among the 586 mothers of infants who are the subject of 3- or 4-month health check-ups

**Fig. 1:**
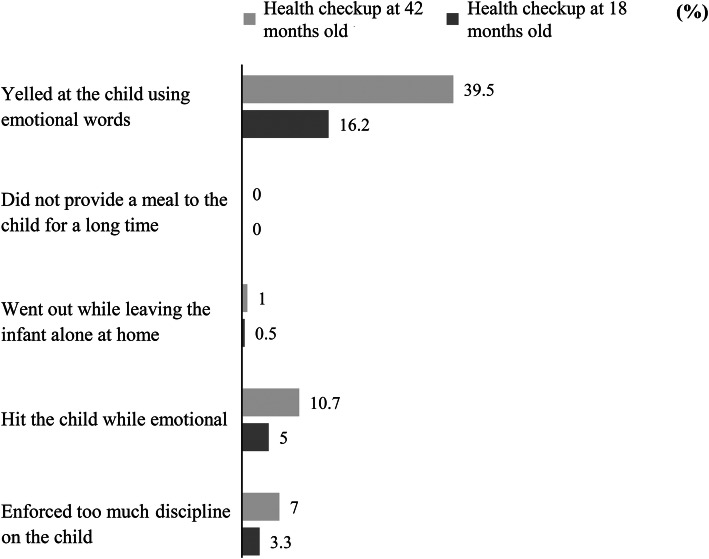
Inappropriate parental behavior (*n* = 582)

Regarding the incidence of inappropriate parental behaviors, 15.7% of mothers inappropriate parental behaviors at both checkups, 28.5% did not have inappropriate parental behaviors during the 18-month-old checkup but did at the 42-month-old checkup, and 51.9% did not have inappropriate parental behaviors at either checkup (Table 2). Among the inappropriate parental behaviors, “yelling using emotional words” was the most common, with 16.2% of the respondents confirming this at the 18-month-old checkup, and 39.5% at the 42-month-old checkup (Fig. 1).

**Table 2:** Mothers’ Inappropriate Parental Behaviors in 18- and 42-Month-Old Health Check-ups

Inappropriate parental behaviors	At 42 months [N(%)^a^]			
	Yes		No	
	*N* = 256		*N* = 322	
At 18 months				
Yes *N* = 113	91	(15.7)	22	(3.8)
No *N* = 465	165	(28.5)	300	(51.9)

^a^ Numbers in four cells add up to 100%

In the 18-month-old checkup, a significant relationship was observed between the mothers’ inappropriate parental behaviors and the characteristics of the mothers and children, namely the birth order of the child, the mother’s feelings in relation to the pregnancy, the mother’s physical and mental health, and interpersonal and financial problems. After adjusting for these variables as covariates, significant relationships were observed between inappropriate parental behaviors and both the child’s birth order (*p* = 0.02) and mother’s interpersonal problems (*p* = 0.04) (Table 3).

**Table 3:** Factors behind the inappropriate parental behaviors of the mothers (at the time of the health check-up at 18 months Old)

Factors^d^	Inappropriate parental behaviors at the time of the health check-up at 18 months^a^				Univariate analysis^c^			Logistic regression		
	Yes		No		OR^b^	95% CI	*P* value	aOR^b^	95% CI	*P* value
	*N* = 114	%	*N* = 468	%						
Child’s sex										
Male	61	19.8	247	80.2	1.03	0.68–1.55	0.89			
Female	53	19.3	221	80.7	1.00					
Order of birth										
First child	64	23.5	208	76.5	1.60	1.06–2.42	0.03^*^	1.66	1.08–2.55	0.02^*^
Second or later	50	16.1	260	83.9	1.00			1.00		
Single or multiple birth										
Single	114	19.8	463	80.2			0.59			
Multiple	0	0.0	5	100.0						
Gestational age at birth										
Less than 37 weeks	5	19.2	21	80.8	0.98	0.36–2.65	0.96			
37 weeks or more	109	19.6	447	80.4	1.00					
Birth weight										
Less than 2500 g	9	20.9	34	79.1	1.09	0.51–2.35	0.81			
2500 g or more	105	19.5	434	80.5	1.00					
Father’s age										
Younger than 30	32	21.1	120	78.9	1.16	0.73–1.84	0.53			
30 or older	79	18.7	343	81.3	1.00					
Mother’s age										
Younger than 30	47	20.5	182	79.5	1.10	0.73–1.67	0.65			
30 or older	67	19.0	286	81.0	1.00					
Mother’s employment										
Employed	52	17.1	252	82.9	0.73	0.48–1.11	0.14			
Not employed	57	22.1	201	77.9	1.00					
Feelings in relation to the pregnancy										
Happy	72	17.3	343	82.7	1.00			1.00		
Unexpected or others	42	25.3	124	74.1	1.61	1.05–2.49	0.03^*^	1.37	0.87–2.17	0.18
Mother’s health										
Good	73	17.2	351	82.8	1.00			1.00		
Easily tired or others	41	25.9	117	74.1	1.69	1.09–2.61	0.02^*^	1.37	0.83–2.27	0.21
Mother’s feelings										
Good	85	17.5	400	82.5	1.00			1.00		
Not good, neutral	29	29.9	68	70.1	2.01	1.23–3.29	0.01^**^	1.41	0.80–2.50	0.24
Family composition										
Nuclear family	96	20.2	380	79.8	1.24	0.71–2.15	0.46			
Extended family	18	17.0	88	83.0	1.00					
Interpersonal problems at home										
Yes	28	30.4	64	69.6	2.07	1.25–3.43	0.01^**^	1.73	1.02–2.95	0.04^*^
No	84	17.4	398	82.6	1.00			1.00		
Financial problems at home										
Yes	19	31.7	41	68.3	2.10	1.17–3.78	0.01^**^	1.50	0.81–2.80	0.20
No	93	18.1	421	81.9	1.00			1.00		

*p < 0.05; **p < 0.01

^a^For some items, the total does not add up to the number in the top row due to missing data

^b^*OR* odds ratio, *aOR* adjusted odds ratio

^c^Analysed using a chi-squared test or Fisher’s exact test

^d^Survey items were collected from the health check-up files for 4 months

The mothers who reported financial problems were 1.61 times more likely to display new inappropriate parental behaviors compared with mothers who didn’t report financial problems (Table 4). Multivariate sub-analysis accounting for financial problems, mothers’ feelings, number of people the mothers could seek support from in regards to childcare only revealed a significant relationship with financial problems (aOR = 2.19, 95% CI 1.13–4.26, *p* = 0.02, data not shown).

**Table 4:** Incidence of mothers’ inappropriate parental behaviors and the associated factors

Factors^d^	Inappropriate parental behaviors^a^ (42-month-old check-up)						
	Yes		No		RR^b^	95% CI	*P* value^c^
	*N* = 165	%	*N* = 300	%			
Child’s sex							
Male	87	35.5	158	64.5	1.00	0.78–1.28	0.99
Female	78	35.5	142	64.5	1.00		
Order of birth							
First child	76	36.5	132	63.5	1.06	0.83–1.35	0.67
Second or later	89	34.6	168	65.4	1.00		
Single or multiple birth							
Single	164	35.7	296	64.3	1.78	0.31–10.33	0.66
Multiple	1	20.0	4	80.0	1.00		
Gestational age at birth							
Less than 37 weeks	6	28.6	15	71.4	0.80	0.41–1.59	0.50
37 weeks or more	159	35.8	285	64.2	1.00		
Birth weight							
Less than 2500 g	9	26.5	25	73.5	0.73	0.41–1.30	0.25
2500 g or more	156	36.2	275	63.8	1.00		
Father’s age							
Younger than 30	44	36.7	76	63.3	1.06	0.81–1.39	0.70
30 or older	118	34.7	222	65.3	1.00		
Mother’s age							
Younger than 30	67	36.8	115	63.2	1.06	0.83–1.36	0.63
30 or older	98	34.6	185	65.4	1.00		
Mother’s employment							
Employed	91	36.4	159	63.6	1.00	0.78–1.28	0.98
Not employed	73	36.5	127	63.5	1.00		
Feelings in relation to the pregnancy							
Happy	119	34.9	222	65.1	1.00		
Unexpected or others	45	36.6	78	63.4	1.05	0.80–1.38	0.74
Mother’s health							
Good	117	33.5	232	66.5	1.00		
Easily tired or others	48	41.4	68	58.6	1.23	0.95–1.60	0.13
Mother’s feelings							
Good	134	33.8	263	66.2	1.00		
Not good, neutral	31	45.6	37	54.4	1.35	1.01–1.81	0.06
Family composition							
Nuclear family	134	35.4	243	64.5	1.01	0.74–1.38	0.96
Extended family	31	35.2	57	64.8	1.00		
Interpersonal problems at home							
Yes	27	42.9	36	57.1	1.23	0.90–1.69	0.22
No	138	34.8	258	65.2	1.00		
Financial problems at home							
Yes	22	55.0	18	45.0	1.61	1.18–2.20	0.01^**^
No	143	34.1	276	65.9	1.00		
Want to continue to raise children in this region? +							
Yes	128	34.6	242	65.4	0.87	0.65–1.16	0.35
Other	37	39.8	56	60.2	1.00		
Whether the father is parenting? +							
Always	102	34.9	190	65.1	0.99	0.76–1.28	0.93
Other	59	35.3	108	64.7	1.00		
Number of people to turn to for advice							
1 or less	13	35.1	24	64.9	0.99	0.63–1.56	0.96
2 or more	152	35.5	276	64.5	1.00		
Number of people to seek cooperation from							
1 or less	22	46.8	25	53.2	1.37	0.98–1.90	0.09
2 or more	143	34.3	274	65.7	1.00		

**p < 0.01

^a^For some items, the total does not add up to the number in the top row due to missing data

^b^*RR* relative risk

^c^Analysed using a chi-squared test or Fisher’s exact test

^d^Survey items were collected from the health check-up files at 4 months. The + indicates items collected at 18 months

There was a significant correlation between maternal age and improvements in inappropriate parental behaviors between the 18- and 42-month-old checkups (*p* = 0.03). Considering the mothers’ parental behaviors, they were divided into two groups based on the Japanese mean age at first birth (30.7 years old in 2018): under 30 and 30 or older (Table 5).

**Table 5:** Improvements in mothers’ inappropriate parental behaviors and associated factors

Factor^c^	Inappropriate parental behaviors ^a^ (42-month-old check-up)				
	Yes		No		*P* value^b^
	*N* = 91	%	*N* = 22	%	
Child’s sex					
Male	48	80.0	12	20.0	1.00
Female	43	81.1	10	18.9	
Order of birth					
First child	54	85.7	9	14.3	0.15
Second or later	37	74.0	12	26.0	
Single or multiple birth					
Single	91	80.5	22	19.5	
Multiple	0	0.0	0	0.0	
Gestational age at delivery					
Less than 37 weeks	5	100.0	0	0.0	0.58
37 weeks or more	86	79.6	22	20.4	
Birth weight					
Less than 2500 g	7	77.7	2	22.2	1.00
2500 g or more	84	80.8	20	19.2	
Father’s age					
Younger than 30	24	75.0	8	25.0	0.42
30 or older	65	83.3	13	16.7	
Mother’s age					
Younger than 30	33	70.2	14	29.8	0.03^*^
30 or older	58	87.9	8	12.1	
Mother’s employment					
Employed	42	82.4	9	17.6	0.81
Not employed	45	78.9	12	21.1	
Feelings in relation to the pregnancy					
Happy	55	77.5	16	22.5	0.33
Unexpected or others	36	85.7	6	14.3	
Mother’s health					
Good	58	80.6	14	19.6	1.00
Easily tired or others	33	80.5	8	19.5	
Mother’s feelings					
Good	68	80.0	17	20.0	1.00
Not good, neutral	23	82.1	5	17.9	
Family composition					
Nuclear family	77	81.1	18	18.9	0.75
Extended family	14	77.8	4	22.2	
Interpersonal problems at home					
Yes	23	85.2	4	14.8	0.78
No	67	79.8	17	20.2	
Financial problems at home					
Yes	16	84.2	3	15.8	1.00
No	74	80.4	18	19.6	
Want to continue to raise children in this region? +					
Yes	67	80.7	16	19.3	1.00
Other	24	80.0	6	20.0	
Whether the father is parenting? +					
Always	49	81.7	11	18.3	1.00
Other	39	81.2	9	18.8	
Number of people to turn to for advice					
1 or less	7	87.5	1	12.5	1.00
2 or more	84	80.0	21	20.0	
Number of people to seek cooperation from					
1 or less	14	100.0	0	0.0	0.07
2 or more	76	77.6	22	22.4	

*p < 0.05

^a^For some items, the total does not add up to the number in the top row due to missing data

^b^Analysed using a chi-squared test or Fisher’s exact test

^c^Survey items were collected from the health check-up files at 4 months. The + indicates items collected at 18 months. Analyzed data only from those who had inappropriate parental behaviors at 18 months

## Discussion

Our analysis of longitudinal municipal data revealed for the first time in Japan that 28.5% of mothers self-reported new inappropriate parental behaviors (as defined by the Japanese national maternal and child health plan) that emerged between the 18- and 42-month-old checkups, while 3.8% decreased their inappropriate parental behaviors. Furthermore, although financial factors impacted whether inappropriate parental behaviors occurred in this time period, maternal age under 30 years was associated with an improvement in these behaviors. These data suggest that incident of and improvements in inappropriate parental behaviors are not inseparable, and the factors that cause these behaviors differ.

The proportion of mothers with inappropriate parental behaviors was 19.6% in the 18-month-old checkup and 44.3% in the 42-month-old checkup. These results are comparable to those reported in the Healthy Parents and Children 21 in 2017 (19.7 and 38.9%, respectively) [32]. As for the frequency of each parenting item, our data was comparable to previously reported numbers. The most common inappropriate parental behavior in our survey was “yelling using emotional words”; 39.5% responded “yes” in 42-month-old checkup. Additionally, 10.7% of mothers reported that they had hit their child when they were feeling emotional during 42-month-old checkup. In a study of parents with a six-year-old child, Isumi, Fujiwara, Nawa, Ochi, and Kato [33] reported that 29.1% responded that they have yelled at their child in a loud voice and 8.2% responded that they sometimes hit their child. In a nationwide study conducted in Japan, Okuzono, Fujiwara, Kato, and Kawachi [34] reported that in the case of children aged 3.5 years, the proportion of parents who spank their child frequently was 10%.

Moreover, 7.0% responded that they enforced too much discipline during the 42-month-old checkup; however, people’s definition of discipline largely differs and it is difficult to differentiate between strict discipline and corporal punishment. Linguistically, the word corporal punishment in Japanese (“taibatsu”) includes guidance at school and discipline at home [21]. However, in Japan, parental behaviors that can be called corporal punishment are now officially a banned form of discipline [34, 35].

Early screening items (at the time of the 4-month-old health checkup) for the prevention of inappropriate parental behaviors are that the child is the first child and that the mother has problems with interpersonal relationships. A woman who is becoming a mother for the first time is susceptible to troubles with childcare and anxiety, and these issues can become risk factors for postpartum depression [36]. Furthermore, interpersonal problems recognized in this study, such as differences in parenting approaches, difficulties in gaining support for childcare, a lack of conversation, and anxiety about how to interact with relatives, are factors that increase stress from relationships with other people or childcare [37].

This study provides insight on the financial problems experienced by the mothers with inappropriate parental behaviors that emerged between the 18- and 42-month-old checkups. Child poverty in Japan (14%) is slightly higher than the OECD average of 12% [38] and it is reported to associate with children’s behavioral problems [39]. Our data indicate a need for financial support for these mothers, which may include employment depending on the situation and the benefits received [40]. There are reports that financial problems heighten parents’ psychological stress and increase the risk of child abuse [33], such that by fulfilling the family’s material demands through financial support, inappropriate parental behaviors can be improved [41]. However, while many previous studies have cited economic poverty as a risk factor for inappropriate parental behaviors and highlighted the need for financial support, there is little evidence describing its effects [42, 43]. In light of these points, the examination of the methods and effects of government services for families requiring financial support is an urgent need. In addition, considering that mothers who did not respond as having a good emotional state tended to have an increased risk of inappropriate parental behavior, a comprehensive system needs to be implemented at the 4-month-old health checkup for mothers who have financial problems. This system should offer the early provision of financial support, together with personal support including childcare consultation services, mental health support to prevent postpartum depression, and social networks for isolated mothers for stronger social bonds to the community [44–46].

In a small number of cases, inappropriate parental behaviors improved leading up to the 42-month-old checkup; this was associated with a maternal age younger than 30 years. In light of the fact that inappropriate parental behaviors in 18-month-old checkup were not related to maternal age and incidence of inappropriate parental behaviors in the 42-month-old checkup were also not associated with maternal age, perhaps younger mothers can more easily improve their behaviors than older mothers. Among the limited parenting reports that focus on parental age, however, one from Australia reported that older mothers were more responsive parents [47]. There are numerous studies on teen parenting and interventions to support them, but further research is needed to confirm whether younger Japanese mothers are more resilient in improving their parenting behaviors.

One limitation of this study was that it used data from the local government’s infant health checkups. As such, there were few items regarding socioeconomic status, i.e., financial status, and educational backgrounds of the subjects were not included. In addition, because the subjects self-reported their inappropriate parental behaviors, we may be underestimating these behaviors. Furthermore, although the concept of inappropriate parental behavior was based on the national policy of “Healthy Parents and Children 21,” this may not be ideal. Nonetheless, the strength of this study was that a longitudinal analysis was performed from the local government’s database, thus facilitating identification of the causes of inappropriate parental behaviors.

## Conclusion

This report suggested that first time motherhood and having interpersonal problems could be early indicators of inappropriate parenting behaviors in the 18-month-old health checkups. In addition, maternal financial problems independently predict new inappropriate parental behaviors that emerge between the 18- and 42-month-old checkups. Maternal financial problems should be included as a screening indicator, and there is an urgent need to consider financial support as an important component of child abuse prevention.

## Data Availability

The datasets generated and analyzed during the current study are not publicly available due to municipal database.

## Abbreviations

FY: Fiscal year
aOR: Adjusted odds ratio
